# Acupotomy in the treatment of tenosynovitis of hand flexor tendons: A systematic review and meta-analysis

**DOI:** 10.1097/MD.0000000000031504

**Published:** 2022-11-11

**Authors:** Dan Li, Xiaole Wang, Ting Fang, Ying Chen, Shanshan Xiang, Junnan Qi, Chengning Liang, Changan Ren, Xiaolan Zhao, Zikai Qiu, Fushui Liu, Xiaojun Yan

**Affiliations:** a Jiangxi University of Chinese Medicine, Nanchang, China; b The Affiliated Hospital of Jiangxi University of Chinese Medicine, Nanchang, China; c The Affiliated Hospital of Shangrao Health School, Shangrao, China.

**Keywords:** acupotomy, meta-analysis, systematic review, tenosynovitis of hand flexor tendons, trigger finger

## Abstract

Acupotomy was used to treat tenosynovitis of hand flexor tendons (THFT) in China. But it’s uncertain about the efficacy of acupotomy for THFT. We plan to evaluate the efficacy and safety about acupotomy therapy in the treatment of THFT through this review.

**Methods:**

The protocol about this review was registered in PROSPERO (registration number: CRD42022330568). We searched 6 databases from their respective inception dates to January 11, 2022. Studies searched was screened by our reviewers, and then the raw data was filtered out. We used RevMan 5.3 software to perform statistical analysis.

**Results:**

11 studies involving 828 patients were shortlisted. The experimental group showed obvious advantages compared with the control group, such as effective rate (odds ratio [OR] = 6.77, 95% CI [confidence intervals] = [3.89, 11.77], *P *< .00001), cure rate (OR = 3.32, 95% CI = [1.81, 6.11], *P* = .0001) and Vas score (MD = −1.21, 95% CI = [−2.00, −0.42], Z = 3.01, *P* < .003).

**Conclusions:**

According to the above results, Acupotomy is an effective and safe treatment for THFT. So it should be recommended for the treatment of THFT patients.

## 1. Introduction

**Figure 1.**
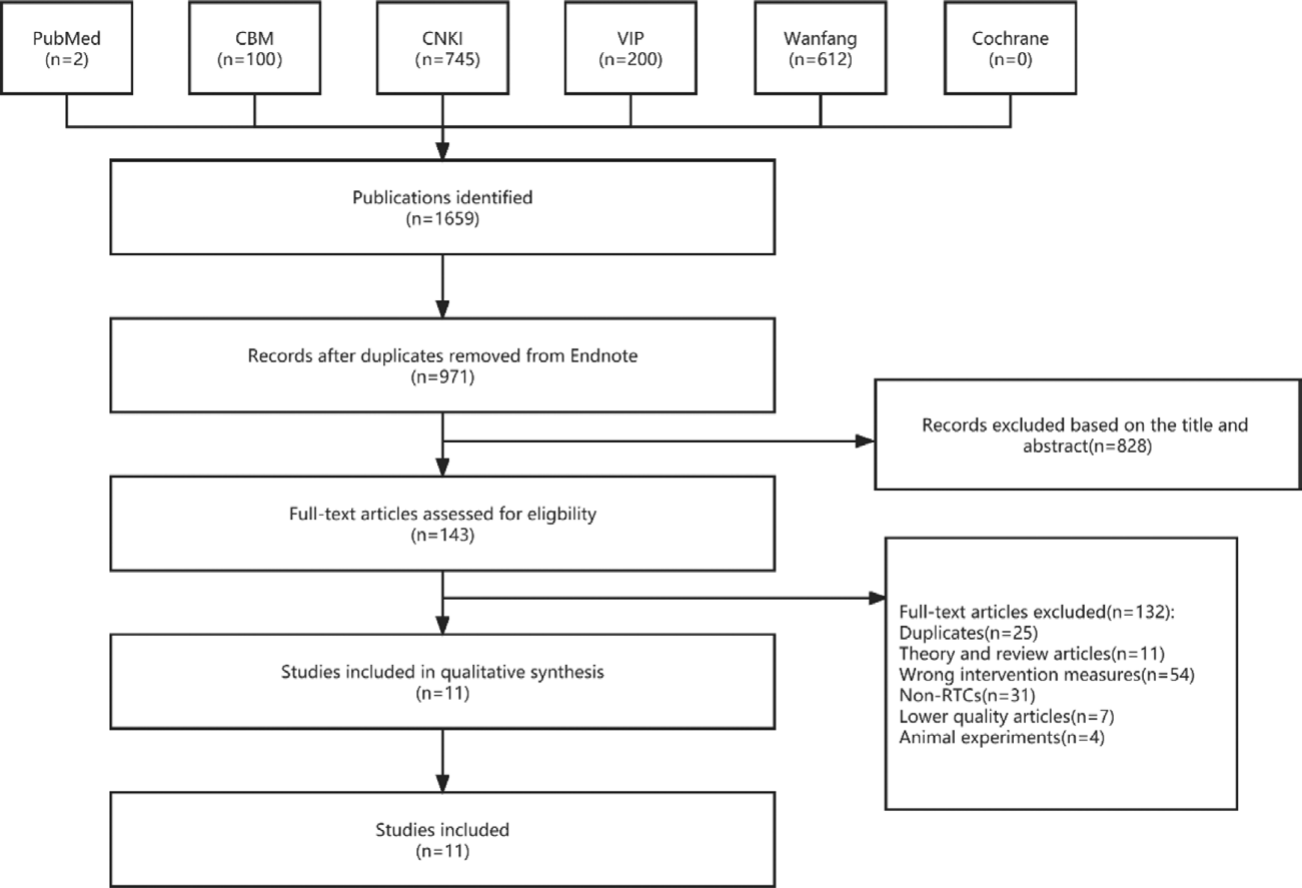
Picture of acupotomy.

**Figure 2.**
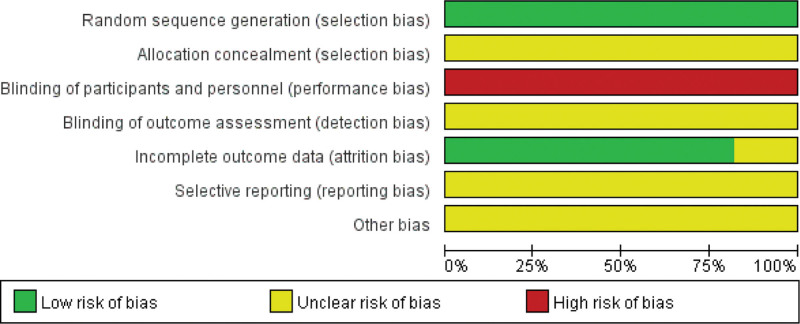
Detail drawing of acupotomy.

Tenosynovitis of hand flexor tendons is a common tendinopathy caused by chronic strain, presenting a narrowing of flexor pulley sheaths (commonly including A1/A2/A3 pulley sheaths) combined with hypertrophy and inflammation of the tendon/sheath interface. Nodular caused by inflammation can be touched, the main clinical features of THFT involve pain, functional impairment. The right hand was more prevalent, as was the dominant hand. The morbidity in women was twice as high as in men.^[1]^ As for treatment of THFT include non-surgical and surgical treatments. Acupotomy invented by Zhu Hanzhang in 1976 is a tool combined acupuncture and knife (Figs. 1 and 2). It pierces into the affected tissue, and then eliminates the adhesion and contracture by cutting and stripping diseased tissue. This type of therapy treats THFT by cutting and stripping thickened swollen sheath pulley. Acupotomy has gained increasing popularity for the management of THFT. However, there is a lack of supportive evidence on the efficacy of acupotomy for THFT. This systematic review aims to provide the available evidence about the efficacy and safety of the acupotomy for THFT.

## 2. Methods

Ethical approval is not required because it is a systematic review. This study was reported in accordance with the Preferred Reporting Items for Systematic Reviews and Meta-Analyses (PRISMA) statement. The protocol was registered in PROSPERO (PROSPERO registration number: CRD42022330568).

### 2.1. Search strategy

We used Chinese and English as search languages. Two reviewers searched literature from 6 databases. As for databases, it includes PubMed, the Chinese Biomedical Literature Database, the China National Knowledge Infrastructure, the China Science and Technology Journal Database, the WanFang databases and the Cochrane library. we retrieved 6 databases from their respective inception dates to January 11, 2022. RCTs were contained, which is related to acupotomy therapy alone or combined with other conventional treatments for THFT.

### 2.2. Inclusion and exclusion criteria

Inclusion criteria include: These trials included were RCTs related to acupotomy for treating THFT; Enrolled patients were not considered nationality, race, age, or sex. As for intervention, the experimental group was treated with acupotomy, and control group was treated with conventional therapy excepting acupotomy. We used Chinese medical efficacy standard (Including effective rate and cure rate) and the visual analogue scale (VAS) to measure outcome; the secondary outcome include adverse events in the acupotomy group and control group to assess safety.

### 2.3. Data extraction

Two reviewers independently extracted raw data from the screened studies, and then cross checked according preordained criteria. We resolved discrepancy through consultation with Fushui Liu. We extracted the key information according following items: first author, year of publication, study location, sample size, baseline characteristics for participants, intervention, randomization method, duration of intervention, allocation concealment, blinding method, follow-up, dropout and withdrawal, outcome measurement indexes, adverse events.

### 2.4. Quality assessment

**Figure 3.**
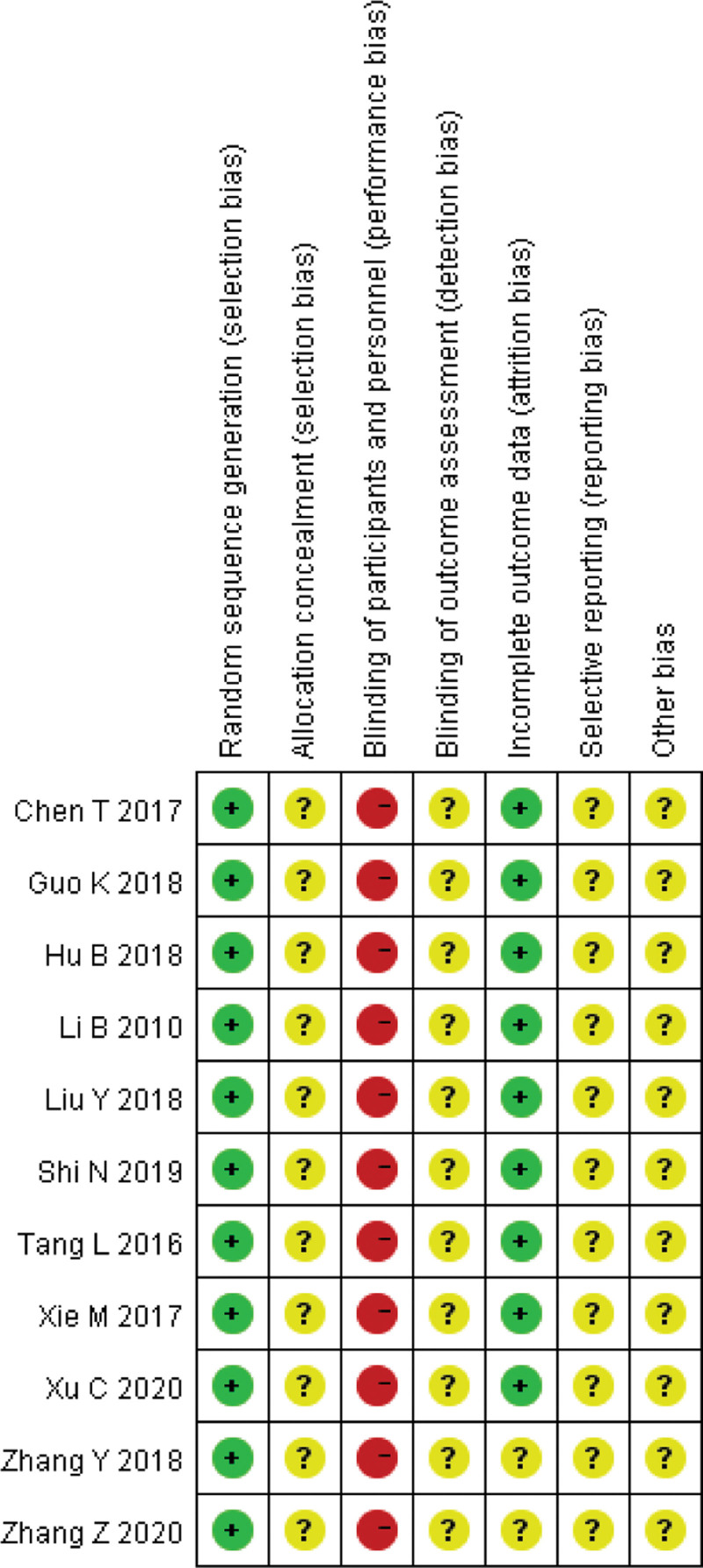
Risk of bias graph.

**Figure 4.**
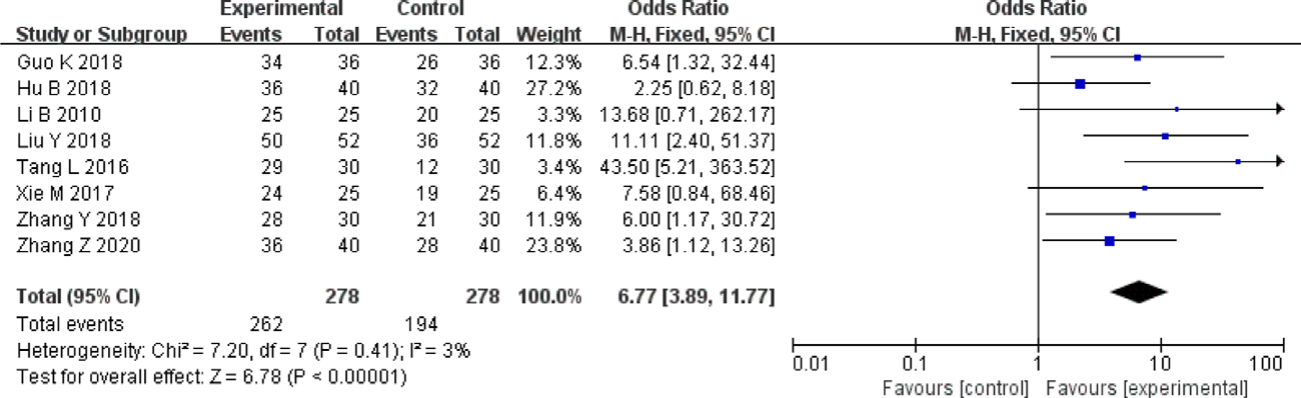
Risk of bias summary.

Our reviewers used the Cochrane Systematic Review Manual (version 5.1.0) RCT bias risk assessment tool to assess the quality and risk of bias (ROB) of the included literature. The ROB results are shown in Figures 3 and 4. If there is some ambiguity we will discuss it to figure out it.

### 2.5. Statistical analysis

The raw data were extracted from the literature that had been screened by reviewers. Relevant raw data were processed by using RevMan 5.3 software. If *P* < 0.05, it is statistically significant. For the categorical data (effective rate, cure rate and adverse events), we calculated combined Odds ratio (OR) with 95% confidence intervals (CI); we estimated combined mean difference (MD) with 95% CI for continuous variables (VAS). Chi-square test and Higgins *I^2^* test was used to analyze heterogeneity between studies, when *I^2^ < *50%, *P > *.10, we used the fixed effect model; otherwise, we applied random effect model.

## 3. Results

### 3.1. Literature search results

**Figure 5.**
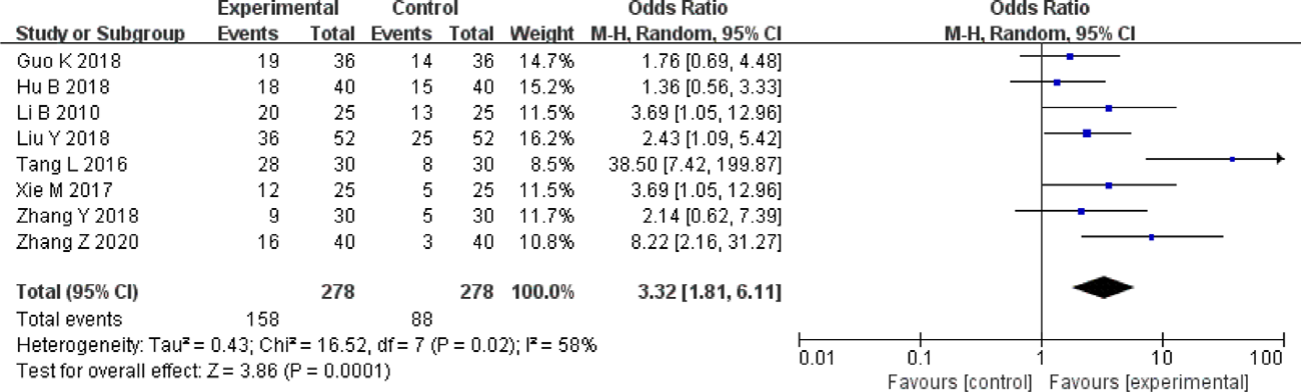
Flow diagram of the study.

According to the above retrieval methods, 1659 literature were obtained. When we used EndNote X7 software to weed out 688 articles it remained 971 studies. We screened the titles and abstracts of remained 971 studies, and then 828 studies were excluded. Finally we scanned full text, 11 RCTs^[2–12]^ were satisfied our inclusion criteria. A total of 11 trials were included. The whole process of study selection is showed in Figure 5.

### 3.2. Quality assessment

Quality and ROB of included trials were assessed by the Cochrane collaboration’s tool. We judged 11 RCTs^[2–12]^ to low ROB on the item of Random Sequence generation. As for the blinding of participants and personnel, all RCTs^[2–12]^ were judged to high ROB as it was impossible to carry out in our included studies. All included studies^[2–12]^ did not refer the blinding of outcome assessment. Nine RCTs^[2–7,10–12]^ were judged low ROB about the incomplete outcome data. Eleven RCTs^[2–12]^ were judged to unclear ROB because all of these protocols of trials conducted in China are not public, so it’s hard to evaluate the item of selective reporting. The ROB results are shown in Figures 3 and 4.

### 3.3. Effective rate

**Figure 6.**
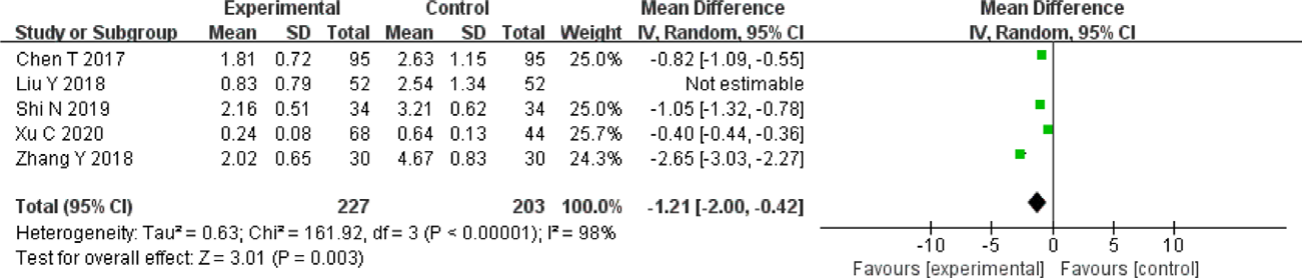
Meta-analysis on the total effective rate of acupuncture versus control group.

Eight RCTs^[2–9]^ reported the effective rate of acupotomy therapy. The result showed that acupotomy therapy was more effective than control group (OR = 6.77, 95%CI = [3.89, 11.77], Z = 6.87, *P *< .00001) in improving effective rate (Fig. 6).

### 3.4. Cure rate

**Figure 7.**
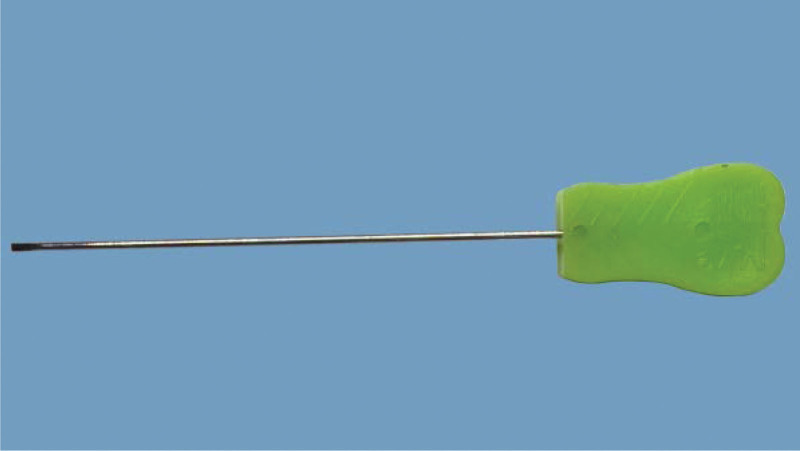
Meta-analysis on the cure rate of acupuncture versus control group.

Eight studies^[2–9]^ reported cure rate of acupotomy. The result showed that acupotomy therapy was more effective than control group (OR = 3.32, 95%CI = [1.81, 6.11], Z = 3.86, *P* = .0001) in improving effective rate (Fig. 7).

### 3.5. VAS score

**Figure 8.**
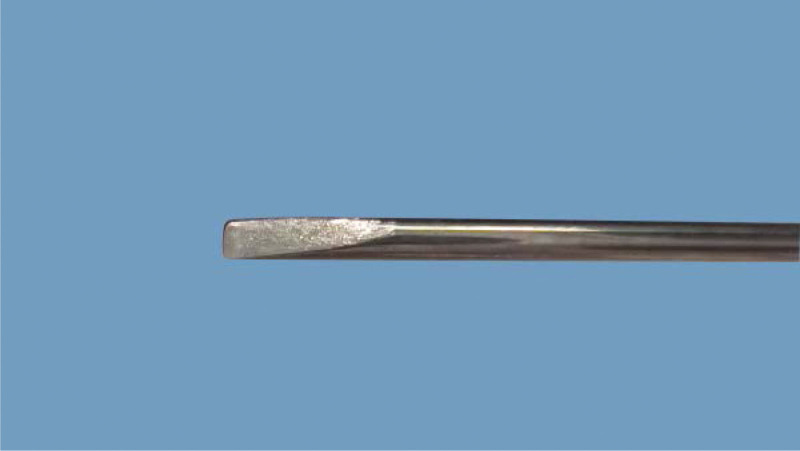
Meta-analysis for VAS score of acupuncture versus control group. VAS = the visual analogue scale.

Five RCTs^[5,8,10–12]^ reported the VAS score of acupotomy therapy. The result showed that acupotomy therapy was more effective than control group (MD = −1.21, 95%CI = [−2.00, −0.42], Z = 3.01, *P* < .003) (Fig. 8).

## 4. Discussion

**Figure 9.**
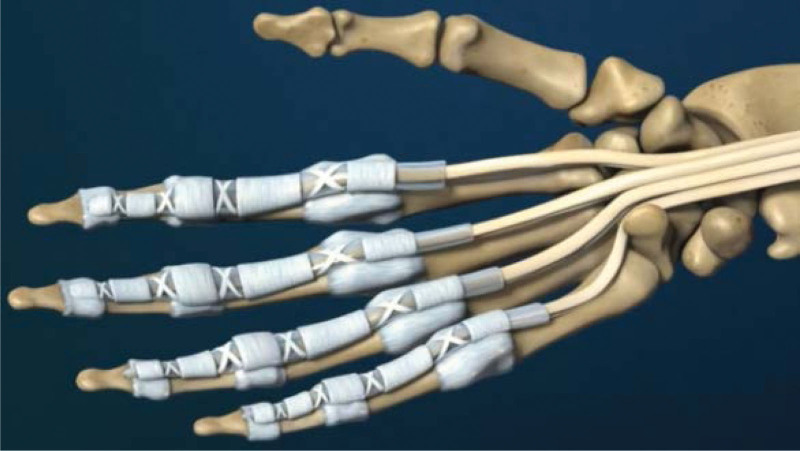
Normal anatomical structure (Cite from Nucleus medical media).

**Figure 10.**
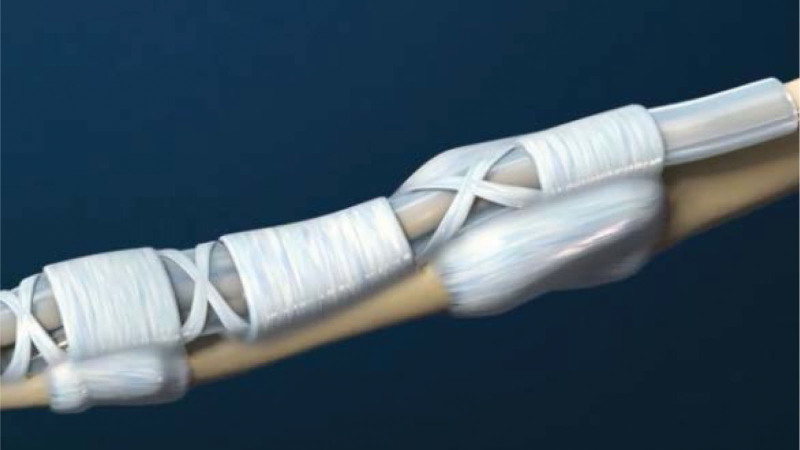
The details of the Normal anatomical structure (Cite from Nucleus medical media).

**Figure 11.**
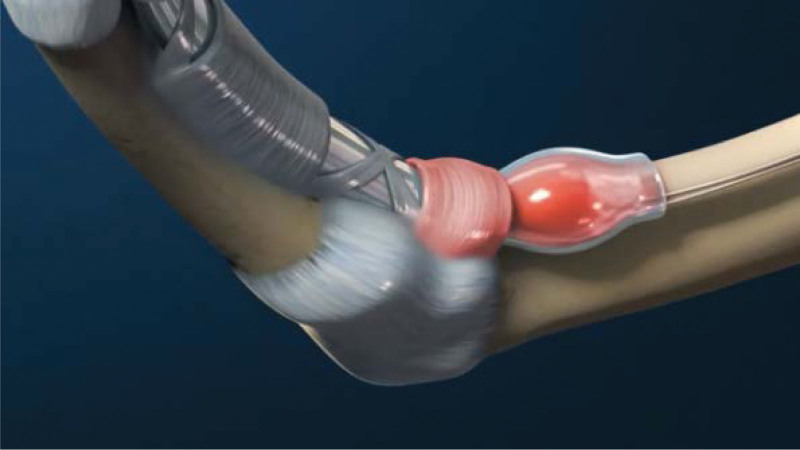
Schematic diagram of pulley lesion (Cite from Nucleus medical media).

**Figure 12.**
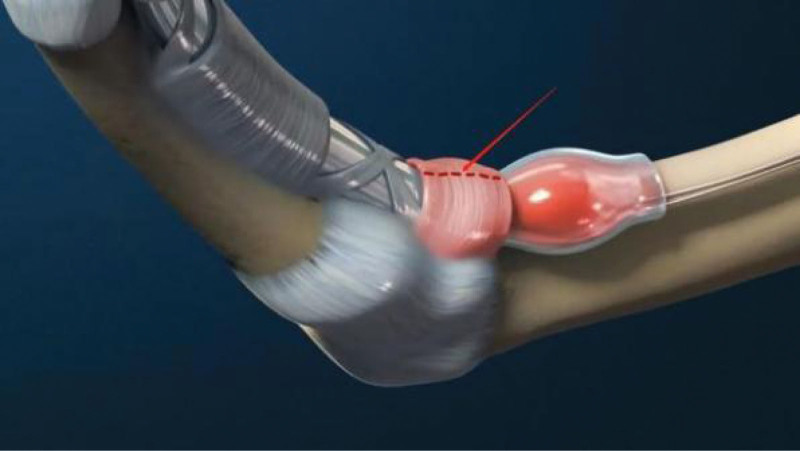
The location that needs to be released (Cite from Nucleus medical media).

**Figure 13.**
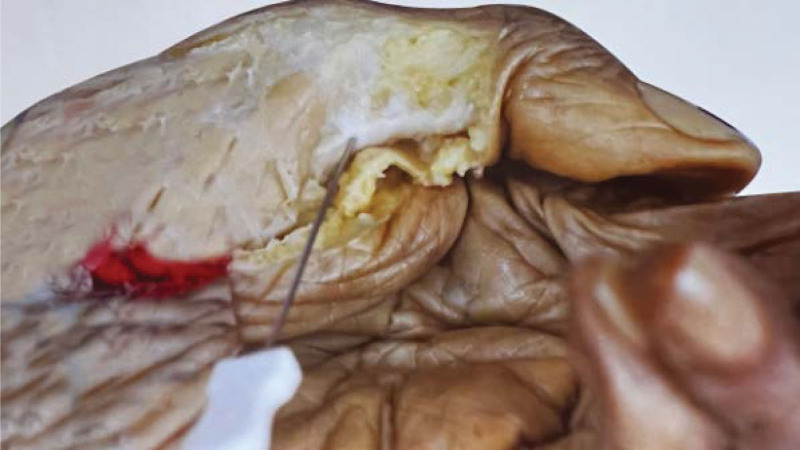
Anatomical diagram of acupotomy treatment (Cite from Shiliang Li. Applied Anatomy and Clinical Practice of Acupotomology).

**Figure 14.**
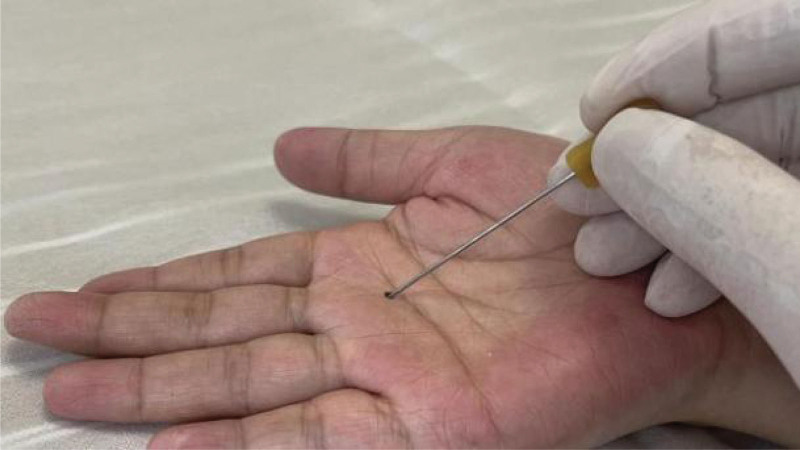
Operation diagram of acupotomy.

**Figure 15.**
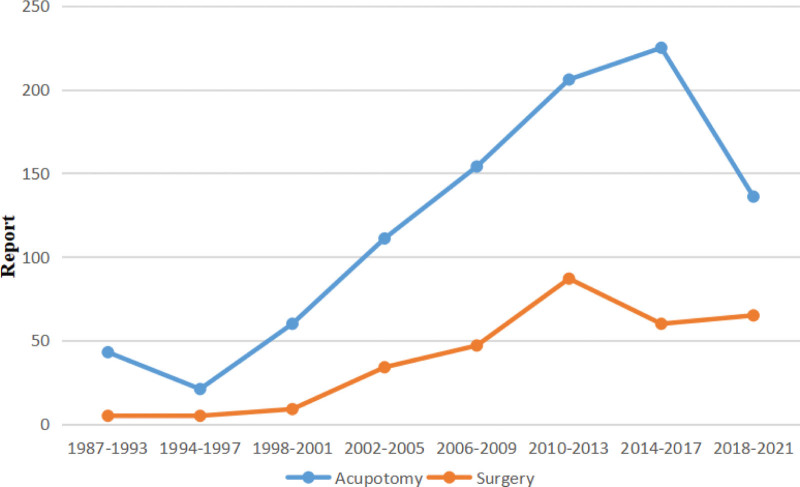
Comparison of the number of surgical and acupotomy reports in CNKI data. CNKI = the China national knowledge infrastructure.

The flexor tendon synovial sheath is a double-layered synovial sheath that surrounds tendon of flexor digitorum superficialis, flexor digitorum profundus tendon and flexor pollicis longus tendon (Figs. 9 and 10). THFT is a common clinical disease, mainly due to frequent finger extension and flexion, tendon and tendon sheath repeated friction strain. It occurs when the gliding movement of the tendon is blocked by the osteofibrous canal of the A1 pulley, preventing the tendon from naturally extending and returning to its initial position^[13]^ (Figs. 11 and 12). These pathogenesis finally result in circulatory disorders, joint popping, pain and dysfunction.^[14]^ It seriously affects hand function and brings great inconvenience to daily life. Most commonly seen in the thumb, followed by the middle and index finger, least in the little finger.^[15,16]^ The symptoms vary from a slight local discomfort to the formation of a tendon blockage, experienced principally in the morning, which leads to a deficit in actively extending the finger, which remains fixed in a flexed position.^[17]^ Although synovial proliferation and fibrosis flexor sheath are identified as triggering factors, there is no consensus in the literature about its true cause and its etiology remains unknown.^[18]^ THFT also appears to be linked to other diseases, such as rheumatoid arthritis, gout, carpal tunnel syndorome, De Quervain’s disease and diabetes.^[19,20]^ Carpal tunnel syndrome is often co-existent with THFT patients, endocrine and metabolic diseases are known to be predisposed to both conditions.^[20]^ At present, conclusive evidences regarding the best treatment option is lacking. There are many therapies for adult THFT, including conservative treatment, such as the method of corticosteroid injection, or surgery, such as open surgery and percutaneous release methods. Although open surgery is an effective method, it is difficult for patients to accept because of its large trauma, long recovery time and high cost. Acupotomy therapy combined traditional Chinese acupuncture treatment with modern surgical principles. This type of treatment works by using a unique instrument which has a needle body with a knife tip of 1mm width to release contracture and eliminate blockage in affected fingers. About the mechanism of acupotomy to treat THFT is still not completely clear. Reported studies showed acupotomy release the thickened tendon sheath, relieve the pressure of the flexor tendon, and help the recovery of the normal metacarpophalangeal joint structure^[21–23]^ (Figs. 13 and 14). The advantages of acupotomy therapy to treat THFT are very obvious for its smaller wound, shorter recovery time and lower costs. The time required for the entire treatment process is less than 1 minute. The whole procedure is not complex, but this type of treatment can be as effective as surgery. The effect of acupotomy therapy in treating THFT patients remains controversial, but clinical trials about acupotomy therapy to treat THFT have shown The Quinnell grade of the experimental group was higher than the control group.^[24]^ Acupotomy therapy to treat THFT is very acceptable and prevalent in Chain. According to current evidence, reports about acupotomy therapy in treating THFT are rising rapidly. By searching the CNKI database, there were significantly more reports of acupotomy therapy in treating THFT than surgery (Fig. 15).

## 5. Conclusions

We pooled the data from 11 studies involving 926 patients. Our pooled analysis indicated that acupotomy therapy was significantly better than control group in improving effective rate, cure rate and VAS score. However, because of the low quality and small sample size of the included studies, high-quality RCTS are needed to confirm our results in the future.

## Author contributions

**Conceptualization:** Ying Chen.

**Investigation:** Zikai Qiu.

**Resources:** Shanshan Xiang, Junnan Qi, Xiaojun Yan.

**Software:** Ting Fang, Ying Chen, Chengning Liang, Changan Ren, Xiaolan Zhao.

**Supervision:** Xiaole Wang.

**Validation:** Fushui Liu.

**Writing – review & editing:** Dan Li.
